# Perceptual Other-Race Training Reduces Implicit Racial Bias

**DOI:** 10.1371/journal.pone.0004215

**Published:** 2009-01-21

**Authors:** Sophie Lebrecht, Lara J. Pierce, Michael J. Tarr, James W. Tanaka

**Affiliations:** 1 Department of Cognitive and Linguistic Sciences, Brown University, Providence, Rhode Island, United States of America; 2 Department of Psychology, University of Victoria, Victoria, British Columbia; Victoria University of Wellington, New Zealand

## Abstract

**Background:**

Implicit racial bias denotes socio-cognitive attitudes towards other-race groups that are exempt from conscious awareness. In parallel, other-race faces are more difficult to differentiate relative to own-race faces – the “Other-Race Effect.” To examine the relationship between these two biases, we trained Caucasian subjects to better individuate other-race faces and measured implicit racial bias for those faces both before and after training.

**Methodology/Principal Findings:**

Two groups of Caucasian subjects were exposed equally to the same African American faces in a training protocol run over 5 sessions. In the individuation condition, subjects learned to discriminate between African American faces. In the categorization condition, subjects learned to categorize faces as African American or not. For both conditions, both pre- and post-training we measured the Other-Race Effect using old-new recognition and implicit racial biases using a novel implicit social measure – the “Affective Lexical Priming Score” (ALPS). Subjects in the individuation condition, but not in the categorization condition, showed improved discrimination of African American faces with training. Concomitantly, subjects in the individuation condition, but not the categorization condition, showed a reduction in their ALPS. Critically, for the individuation condition only, the degree to which an individual subject's ALPS decreased was significantly correlated with the degree of improvement that subject showed in their ability to differentiate African American faces.

**Conclusions/Significance:**

Our results establish a causal link between the Other-Race Effect and implicit racial bias. We demonstrate that training that ameliorates the perceptual Other-Race Effect also reduces socio-cognitive implicit racial bias. These findings suggest that implicit racial biases are multifaceted, and include malleable perceptual skills that can be modified with relatively little training.

## Introduction

Many human attitudes arise automatically and without conscious control. Such attitudes, termed “implicit biases,” manifest in a variety of social domains [1]. For example, people prefer the young to the old, and pair women with the home more often than they pair women with the laboratory [2]. People show a negative implicit association with members of a racial group other than their own [3], [4]. Critically, this sort of implicit bias *does not* correlate with explicit judgments of race – what we say we believe [3]. Moreover, this dissociation is not simply a laboratory phenomenon. The 2008 United States presidential election has rekindled discussion of the “Bradley effect,” in which voters' public preferences in pre-election polls do not necessarily correlate with their implicit judgments, as measured by their actual private votes for African American candidates [5], [6]. As such, explaining the origins and potential plasticity of implicit racial biases could aid our understanding of the interaction between human cognitive and social processing, *and* help address real-world social biases.

Our thesis is that implicit racial bias is comprised of both social and perceptual components. In conjunction with measured social biases for unfamiliar faces belonging to different racial groups, behavioral evidence suggests that people perceived other-race faces as more similar than own-race faces [7], [8] a bias termed the Other-Race Effect (ORE). By far the most widely accepted account of the ORE is experiential. That is, through one's lifespan, greater experience with faces of a particular race leads to greater expertise in individuating faces of that race. This explanation is bolstered by several recent studies that indicate that exposure to a specific race face morphology early in life equips people to better individuate faces of that race later in life, regardless of the learners' own-race. For example, African infants adopted into Caucasian families show an ORE favoring Caucasian faces akin to that exhibited by Caucasian children [9] and Chinese infants show an ORE favoring Asian faces over African, Caucasian and Middle Eastern faces [10].

Here we investigated whether the social and perceptual components of implicit biases affect one another. We conjectured that observers are less facile at making individual social attributions for those faces that they are less able to differentiate, and therefore show more social stereotyping. In light of the evidence that the ORE is a perceptual expertise effect for own-race faces, meaning subjects demonstrate subordinate level expert recognition for their own-race faces and basic level recognition for other-race faces; we further predicted that “expertise training” [11] with other-race faces – a procedure that improves observers' ability to individuate objects within the training domain – should reduce or ameliorate the degree to which other-race faces are stereotyped. That is, given an increased ability to assign individual social attributes to individual other-race faces, we predict a concomitant reduction in measured implicit racial biases. In sum, following perceptual expertise training for other-race faces, we expect subjects to show a reduction in their implicit racial bias that is correlated with their improvement in differentiation of other-race faces, also termed improvement in perceptual individuation.

To evaluate our prediction Caucasian subjects were trained on African American faces. A baseline of each subject's ability to individuate African American and Chinese faces was obtained using an old-new recognition memory task with the two races intermixed across trials. Similarly, a baseline of each subject's implicit racial biases were obtained by measuring an “Affective Lexical Priming Score” (“ALPS”). This measure is similar to the “Implicit Association Test” (“IAT”; [3]) and “Bonafide Pipeline” (“BFP”; [4]), tests which have significantly advanced our understanding of the behavioral and neural [12] underpinnings of implicit racial biases. The IAT probes implicit biases by activating competing or complementary semantic categories in long-term memory. However, because we are interested in studying the relationship between other-race perceptual and implicit social biases, we modeled ALPS more on the BFP [4] which probes implicit biases using perceptual primes (e.g., face pictures) as opposed to semantic constructs (e.g., the categories “good” and “bad”). When the BFP is modified to be less perceptual and more categorical there is better consistency between the BFP and the IAT [13].

**Figure 1:**
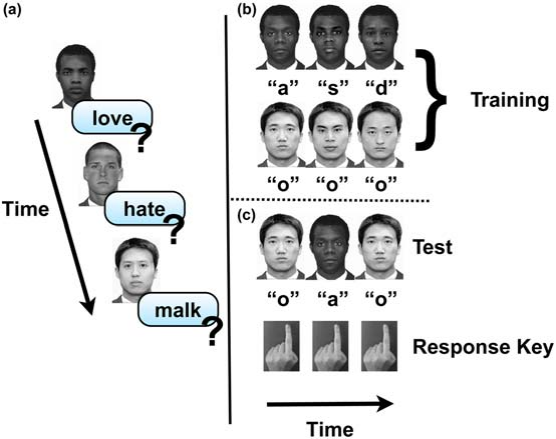
Experimental design for ALPS (a) depicting three consecutive trials where subjects are presented with a face prime, followed by a letter-string to which they respond word or non-word. In the individuation training condition (b) subjects learn to associate 8 individual African American faces with particular letters, whilst simultaneously categorizing Chinese faces by pressing “o”. At test (c) subjects recall the letter learned for each African American individual, but respond “o” for all Chinese faces.

ALPS works by presenting subjects with a face image followed by a letter-string and instructing them to judge whether the letter-string denotes a valid, English word (Figure 1a). The letter-string can be of positive, negative, or neutral valence, or a nonsense, non-word (e.g., “love” “hate” “tree” and “malk”). ALPS changes the task from an explicit word valence judgment in the BFP to a lexical decision judgment, where valence processing is implicit and task irrelevant. As such, ALPS is more likely to be sensitive to purely implicit biases. Thus, we are reasonably confident that ALPS is at least as effective as other tools for assessing implicit associations.

To ensure that any post-training changes in implicit bias arise from improved individuation of other-race faces, rather than from general experience, subjects were randomly assigned to one of two training conditions, individuation or categorization [11]. In both conditions, subjects saw an equal number of other-race faces. In the individuation condition (Figure 1b), subjects underwent perceptual expertise training with African American faces [14]. In the categorization condition, subjects categorized each face as Chinese or African American. Following training, we repeated both measures and assessed any change in performance.

We predicted that if subjects hold negative associations towards African American faces, in ALPS trials containing an African American face and a negative word (congruent trials) subjects response times will be fast; conversely, in ALPS trials containing an African American face and a positive word (incongruent trials) subjects response times will be slow. In contrast, following perceptual expertise training, we predicted that each subject's response time difference between congruent and incongruent ALPS trials would be reduced in proportion to any reduction of their ORE. That is, implicit racial biases are ameliorated because associations between negative valence words and African American faces are weakened; consequently, African American faces show reduced priming for lexical decisions of negative words.

## Results

Our results confirm these predictions. Prior to training, all subjects took longer to respond to positive words preceded by an African American face than they did to respond to negative words preceded by an African American face, *t*(19)  =  2.27, *p*<0.01 (all *t*'s one-tailed). For subjects in the individuation training condition, post training this response time difference between is no longer significant, *t*(9)  =  0.232, *p*>0.4; in contrast, for subjects in the categorization training condition, post training this difference remains significant, *t*(9)  =  1.81, *p*<0.05.

The results for perceptual training show a main effect of training *F*(1, 19)  =  17.31, *p*<0.01 demonstrating that all subjects improve with training. The interaction between training and training condition (individuation/categorization) is marginally significant *F*(1, 19)  =  3.44, *p*  =  0.079 and equivalent to the behavioral training effect reported in [15]. Prior to training there are no significant differences between the two levels of training groups, *t*(38)  =  0.54, *p*>0.59 (all *t*-tests reported for perceptual effects are two-tailed independent tests). Post training there is a significant difference between the two groups, *t*(38)  =  2.18, *p*<0.03, indicating that subjects in the individuation condition are better at individuating faces post training as compared to subjects in the categorization condition.

**Figure 2:**
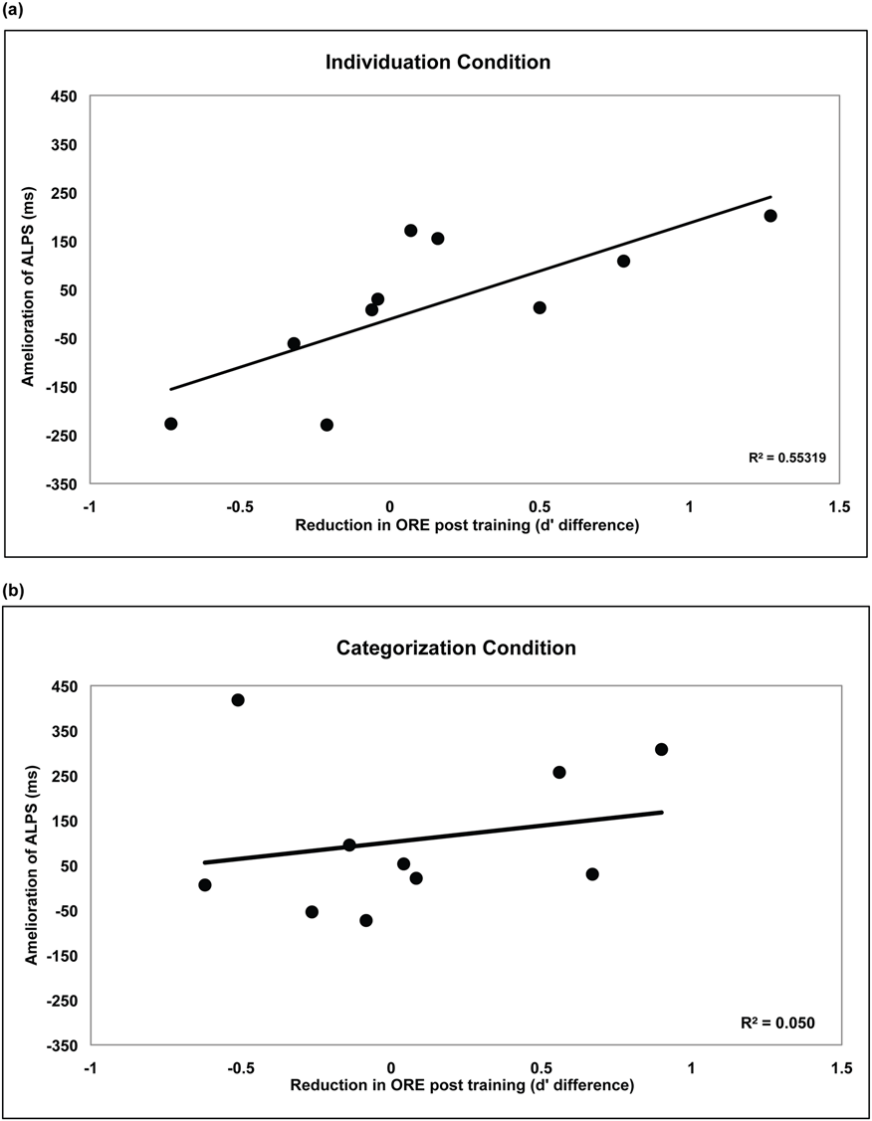
Amelioration of ALPS as a function of a reduced ORE for the individuation condition (a), and the categorization condition (b). The amelioration of ALPS is measured as the RT difference for Pre-Training([African American face with positive word] – [African American face with negative word]) – Post-Training([African American face with positive word] – [African American face with negative word]). As such, an increasing numbers on the Y-axis indicate an increasing *reduction* in racial bias. Reduction in ORE post training is measured by Post-Training(ORE d' score) – Pre-Training(ORE d' score).

It is worth noting that we are not claiming these training effects can account for the multitude of factors contributing to implicit racial biases; instead, we are focusing on the possible contributions one factor – perceptual biases. It is also important to note that when assessing implicit social biases, group means are often difficult to interpret without an extremely large sample size [2]. Given our relatively small sample, we gain a better understanding of the relationship between perceptual and social biases by correlating the change in each individual's ORE with their change in implicit race bias. Across subjects we expect that individuals showing the largest reduction in their ORE will also show the largest reduction in their implicit racial bias. For both training conditions, we correlated each individual's change in ORE for African American faces with their reduction in ALPS. In the individuation training condition, post-training, a reduction in ORE correlated with a reduction in implicit racial bias, *r* ^2^ (8)  =  0.55, *F*(1,9)  =  9.90, *p*<0.01 (Figure 2a). In contrast, in the categorization training condition there is no significant correlation, *r* ^2^ (8)  =  0.05, *F*(1,9)  =  0.42, *p*>0.5 (Figure 2b). Thus, the positive correlation between perceptual training and reduction in implicit social bias is not due to simple exposure to African American faces, nor a result of repeating either the ORE or ALPS tasks. Put succinctly, as Caucasian observers become better able to individuate African American faces, their implicit racial biases are reduced.

## Discussion

Our study addresses the origins and malleability of implicit social biases, in particular, those that manifest as racial stereotyping. Although there has been extensive study of both implicit racial biases and asymmetries in the perceptual processing of own- and other-race faces, the two lines of research have rarely been connected (although see [16]). Interestingly, in the few studies that have attempted to make some connection, they typically attempt to account for the ORE by the presence of implicit racial biases [17], [18] usually on the basis of significant correlations (with *r*'s ranging from .47 to .64) between a individual subjects' ability to individuate own-race versus other-race faces and the same subjects' IAT scores for own-race versus other-race comparisons [19]. To us, drawing the causal arrow in this direction or in *any* direction based on simple correlations seems problematic. First, perceptual systems serve as the input to socio-cognitive systems, not the other way around. Second, although perceptual systems are subject to top-down influences [20], it is unclear whether such feedback can change one's visual discrimination ability; more likely, any top-down effects will shift one's perceptual biases, but not our actual ability to more or less effectively distinguish between two objects [21]. Third, simple correlations between these two measured effects do not demonstrate causality; for example, it may be that greater exposure to other-race faces ameliorates both the ORE and implicit racial biases, but without the two effects being directly related.

More often than not, implicit racial biases have been treated as purely socio-cognitive in nature. In contrast, the data presented here implicates visual face recognition processes as one component of implicit racial biases and suggests that, to some extent, failure to perceptually individuate between members of an other-race group leads to fewer social distinctions between members of that group. Supporting this conclusion, plasticity in those visual processes responsible for individuating between faces within a race, that is, the perceptual mechanisms underlying the other-race effect, produces concomitant changes in our subjects' measured implicit racial bias. More broadly, this finding implies that our perception of and cognitive response to other-race individuals is not a product of a one-dimensional social attitude, but rather a multi-dimensional response comprised of social, perceptual and cognitive properties.

It should be acknowledged, however, that our interpretation of these novel results is related to a specific definition of what constitutes stereotyping. More specifically, a stereotype has been described as the “lumping together of unknown individuals” [22]. From a perceptual perspective, “lumping together” is the operational definition of the ORE, which functionally manifests as a poorer ability to individuate other-race faces. Given different levels of perceptual individuation between own- and other-race faces, it is reasonable to assume that, as the input to our socio-cognitive system, this difference in the granularity of visual categorization will likewise affect the granularity of social categorization processes. Put more colloquially, perceptual “lumping together” prompts social “lumping together.” And once stereotypes, positive or negative, are associated with some members of a social group, the fact that individuals within that group are less differentiated both perceptually and socially makes it more likely that these biases will be extended to all of its members.

Beyond arguing for the inclusion of perceptual factors in how we understand the creation and application of implicit social biases, our results leverage the plasticity of the perceptual categorization system, or, as it is sometimes referred to, “perceptual expertise” [14]. That is, given our understanding of how experience shapes vision, amelioration of the ORE seems likely with massed training on other-race faces. The results presented here, in conjunction with social measures, and in Tanaka et al. [15] with perceptual measures, confirm this prediction. Critically, it is not simply that we find a correlation between one's measured ORE and one's measured implicit racial bias, but rather, that we see correlated *changes* in the latter with the former. One way to better understand how individuation training ameliorates implicit racial bias is to consider the neural changes that are likely taking place as a consequence of such training. With respect to the neural representation of faces, neuroimaging reveals that within the fusiform region associated with face processing [23]; Caucasian subjects show a larger neural response to Caucasian faces than they do in response to African American faces [24]. Undoubtedly, this difference in neural coding has implications for the nature of the visual inputs passed to other cognitive systems, in particular, those brain mechanisms involved in the social representation of faces [25]. The specific brain regions associated with social processing include the amygdala, found to have heightened response for African American faces as compared to Caucasian faces [26], and the nucleus accumbens associated with positive reappraisal and reward [27]. What is at present unanswered, is how “perceptual expertise” training affects these non-visual areas of the brain. Under one account, the only training-associated changes in these areas are those arising from changes in the input coming from high-level visual areas. Alternatively, visual differentiation may directly impact the neural codes for social categorization, much as lateral prefrontal cortex can “drive” visual categorization and, potentially, feedback to visual areas [28]. Here it is possible that training-induced changes in the coding of objects within the middle fusiform gyrus [14], in turn lead to changes in the signal of limbic or regions of pre-frontal cortex (most likely ventral lateral regions), for example, the amygdala, which, ultimately, has consequences for social behaviors.

In sum, our results establish two novel theoretical claims about implicit social biases and stereotyping behavior. First, these behaviors are multidimensional, emerging from both socio-cognitive functional systems and from perceptual categorization processes. Second, these behaviors are malleable, and, critically, can be altered by “perceptual expertise” training that has direct implications for how effectively observers can individuate between members within a race. Moreover, experience alone does not seem sufficient to produce changes in other-race face recognition or implicit social bias. That is, beyond simple exposure, improvements in other-race face recognition appear to be precipitated by the motivation to process faces at the individual, rather than the group, level. Here we show that such training also has indirect implications for how observers end up processing and ascribing group-general stereotypes to that race. As such, our findings have great potential for how we understand and address the real-world consequences of racial stereotyping.

## Materials and Methods

Training methods were similar to those used by [15]

### Subjects

Twenty volunteers participated in our study: 10 in the individuation condition and 10 in the categorization condition. Subjects were recruited from the University of Victoria campus and the local community. All subjects were Caucasian as established by self-report through a written questionnaire completed by each subject prior to participating in the study (see Questionnaire S1). All subjects gave informed, written consent before beginning the study. This study was conducted in accordance with and approved by the Human Ethics Committee at the University of Victoria, British Columbia.

### Stimuli

Stimuli consisted of 264 gray-scaled images of African American, Chinese, and Caucasian (used for category verification tasks, see procedure) individuals created in Adobe Photoshop (San Jose, CA). African American and Caucasian faces were obtained from the Department of Corrections public face databases from the states of Florida, Arkansas, Georgia and Kansas. Chinese faces were obtained from the CAS-PEAL face database (ICT-ISVISION Joint Research and Development Laboratory for face recognition, sponsored by National Hi-Tech Program and ISVISION Technologies Co., Ltd.). All faces were male, between 20–35 years of age, and exhibited neutral expressions. The race of each stimulus face was determined through visually-based ratings as provided by seven raters working in the Department of Psychology at the University of Victoria – only those face images for which there was consensus across all raters were used as stimuli. Internal face features were removed from the original images and placed digitally into a standard face template, which held constant hairstyle, clothing, and face contour. Luminance was controlled within each racial group (African American  =  121 mean luminance, Chinese  =  145 mean luminance, Caucasian  =  161 mean luminance). Images were presented on a computer monitor (LG monitor, 1024×768 pixel screen resolution) 60 cm from the subject and were formatted as 225×311 pixel bitmaps. They subtended a visual angle of approximately 6.65 degrees (vertical) and 7.59 degrees (horizontal).

The letter-strings presented in ALPS were a combination of words (72) and non-words (72). Of the words presented, 24 were positive, 24 negative, and the remaining 24 were neutral. The positive and negative words were a combination of words from the IAT [3], the BFP [4], and an affective word website: http://winspiration.com. The neutral words and the non-words were generated exclusively for this experiment. Kucera-Francais written word frequency were determined for all words by the MRC Psycholinguistic database. The words were then categorized as: low frequency defined as less than or equal to 50 words per million, medium frequency defined as 51–100 words per million, or high frequency defined as 100+ words per million. The non-words were a combination of letters that did not form a word in written or spoken English; however, the non-words did follow the conventions of English grammar and were therefore pronounceable. Word length was determined for all word categories and matched across word and non-word conditions. The letter-strings were presented in white Times New Roman font on a 50% gray background.

### Procedure

#### Pre-Assessment (Memory Task)

Preceding training, subjects completed a perceptual old/new task, consisting of three blocks, divided into study and test phases. During the study phase subjects viewed random presentations of 24 study faces, 12 Chinese and 12 African American, which they were instructed to memorize for a subsequent recognition test. Trials began with a 250 ms fixation cross, followed by the presentation of the face stimulus for 2000 ms, with a 1000 ms ISI. During the test phase subjects viewed 48 faces, 24 of which were present during the study phase, and 24 of which were novel. Subjects made an old/new judgment pressing a keyboard button upon the presentation of each face. Faces displayed for 3000 ms, and subjects responded during this, or 2000 ms following. This procedure was repeated for three blocks separated by rest breaks.

#### Pre-Assessment (ALPS)

Following the memory task, and prior to training, subjects completed ALPS, a measure of implicit racial bias (Figure 1a). In this task, subjects were presented with a visual prime presented on a computer screen for a duration of 250(ms) that was either a Caucasian, Chinese, or African American face. After a brief Signal Onset Asynchrony (SOA) of 200(ms), subjects were then presented with a letter-string located in the centre of the screen. The subjects' task was to determine whether the letter-string was a word or a non-word. Once the subjects responded there was a 1 second inter-trial interval (ITI) after which the next trial began. There were a total of 144 trials, which were divided equally by the three race primes. Of the 144 trials 50% were non-word trials. The word trials were further divided equally into positive, negative, and neutral word trials. The experiment was randomized such that the face – letter-string pairing varied across subject and therefore across pre-and post assessments.

#### Behavioral Training

Following the pre-assessment, subjects took part in five sessions of behavioral training, each lasting approximately 45 minutes. Training began the day following the pre-training assessment and ran every second day until the post-training assessment. Subjects were randomly assigned to either individuate African American faces while categorizing Chinese faces, or to individuate Chinese faces while categorizing African American faces. It is important to note that all subjects saw exactly the same visual stimuli, however the level at which they were discriminated differed across conditions. Each session involved completing a learning task, a naming task, and three category verification tasks, and through this procedure subjects learned to individuate eight novel faces of Chinese or African American ethnicity per day, while categorizing eight novel faces of the converse.

#### Learning Task

To begin the first training block, 2 Chinese and 2 African American faces with corresponding labels underneath were displayed in random order for subjects. During the remaining training blocks 1 new face from each race was displayed, until all 8 faces from each race were learned. In the individuation condition, a label of either “a”, “s”, “d”, “f”, “g”, “h”, “j”, or “k” was displayed below the stimulus. In the categorization condition the label presented was always the letter “O”. All faces were introduced two times in this manner, and subjects were to respond with the corresponding key on the keyboard. Stimuli were displayed until subjects responded, with 1000 ms between stimulus presentations (Figure 1b).

#### Naming Task

Following each block of learning, subjects completed a naming task, during which the stimuli from the learning task were randomly presented, without a label, for 3000 ms. Subjects responded by pressing the button corresponding to the previously learned identity (Figure 1c). Feedback followed, with corrective feedback given on incorrect trials. In order to continue to the next block, subjects were required to label the faces with 100% accuracy, and the block repeated until they were successful. Subjects proceeded until they had learned to individuate all 8 faces from their trained race, and categorize all 8 faces from their untrained race.

#### Category Verification Tasks

Following 7 blocks of learning and training tasks, after subjects had successfully learned to individuate all 8 faces within their trained race, and categorize all 8 faces from their untrained race, three category verification tasks were performed, using similar methods to those used by Scott et al. (2006). During the first task a label (e.g. “Chinese” or “A”) was presented for 500 ms, followed by a fixation cross for 250 ms, and then a face for 500 ms. Subjects had to respond with a button press as to whether or not the label matched the image. For their trained race the image had to be an exact match, whereas in their untrained race a same trial was considered any face from the same race. Different trials for the trained race consisted of a different face within the same race, while different trials for the untrained race were previously unseen Caucasian faces. Half of the trials were same trials, and half were different. Feedback was then presented for 500 ms following the response. Next subjects completed a reverse category verification task with identical procedures with the exception that the label and the image switched positions. Finally a speeded category verification task was performed. Procedures were identical to the original category verification task except subjects had a deadline of 1000 ms to respond. Subjects completed 48 trials for each task, seeing each face twice.

#### Post-Assessment (Memory Task and ALPS)

On the day following, all five behavioral training sessions subjects completed the post-assessment memory task and the ALPS task with procedures and stimuli identical to those used during pre-assessment.

## Supporting Information

Questionnaire S1 Written questionnaire completed by each subject prior to participating in the study.
(0.03 MB PDF)
