# Supplementary material for: Perceptual Other-Race Training Reduces Implicit Racial Bias
Source: PLoS One. 2009 Jan 21;4(1):e4215. doi: 10.1371/journal.pone.0004215 (PMC2627769; doi:10.1371/journal.pone.0004215)
Supplement: Questionnaire S1 — Written questionnaire completed by each subject prior to participating in the study. (0.03 MB PDF) [file pone.0004215.s001.pdf]

Participant No.________

Background Questionnaire

1. How old are you? ______

2. What is your ethnicity? (Please circle one)

African	Chinese
Hispanic	East Indian
Caucasian	Other (please specify) ____________________

3. In which country were you born? ________________________________

4. Have you ever lived in a country other than your place of birth? Please circle Y / N

If yes, please list:

Country	Length of time (approximately)
________________________________	________________________________
________________________________	________________________________
________________________________	________________________________
________________________________	________________________________

5. Where was your mother born? ________________________________

6. What is her ethnicity? ________________________________

7. Where was your father born? ________________________________

8. What is his ethnicity? ________________________________

9. Do you have any relatives (by birth or marriage) or close friends who are members of other ethnic or racial groups? Please circle Y / N

If yes, please list:

Their ethnicity	Relationship to you	How often you see them (circle one)
_____________	____________________	Daily Weekly Yearly <Yearly
_____________	____________________	Daily Weekly Yearly <Yearly
_____________	____________________	Daily Weekly Yearly <Yearly
_____________	____________________	Daily Weekly Yearly <Yearly

10. Have you ever lived with people from other ethnic groups? Please circle Y / N

If yes, please list:

Their ethnicity	Length of cohabitation	Your age when you moved in
_____________	____________________	____________________
_____________	____________________	____________________
_____________	____________________	____________________
_____________	____________________	____________________

THANK YOU FOR YOUR PARTICIPATION!
